# Redefining CMR reference standards through prognostic validation

**DOI:** 10.1016/j.jocmr.2025.101970

**Published:** 2025-10-11

**Authors:** Zahra Raisi-Estabragh, Matthias G. Friedrich

**Affiliations:** Centre for Advanced Cardiovascular Imaging, William Harvey Research Institute, Queen Mary University of London, NIHR Barts Biomedical Research Centre, Barts Heart Centre, Barts Health NHS Trust, London, UK; Department of Medicine and Diagnostic Radiology, McGill University, Montreal, Quebec, Canada

Cardiovascular magnetic resonance (CMR) is the gold-standard for reproducible assessment of myocardial tissue composition, morphology, and function. The boundaries of health and often the severity of disease are based on CMR reference ranges defined in healthy cohorts.

However, healthy adult hearts vary considerably by age, sex, and ethnicity. The cardiovascular phenotype is also importantly influenced by environmental and genetic exposures – which may exert healthy or unhealthy remodeling patterns, without necessarily causing overt cardiovascular disease. Such phenotypic features are quantified by metrics derived from CMR, with variations in reference ranges depending on the composition of the underlying cohort. There is now general agreement that best practice requires stratification of reference ranges by demographic factors. However, there is no consensus on the definition of cardiovascular health. Some researchers have gone to great lengths to exclude individuals with any disease or risk factors from their reference samples, often conducting investigations to detect undiagnosed and pre-clinical disease. Others have argued that such approaches create atypical “hyper-healthy” samples that are inappropriate for creating reference ranges intended for unselected clinical cohorts.

Technical factors related to scanner features, acquisition parameters, post-processing software and image analysis methods are other major sources of variation in reference ranges, with the latter being the most influential. There is currently no single universally accepted standard for contouring methodology, with differences in practice within and across centers. A key variation is whether intracavity trabeculae and papillary muscles are included (anatomic) or excluded (smooth) from the left ventricular (LV) mass when drawing endocardial ventricular contours. Both approaches are commonly used in clinical practice, with smooth segmentation thought to be more reproducible and anatomic segmentation proposed as providing a more accurate anatomic representation. Other variations include differences in the cardiac phase at which LV mass is measured – while traditionally this is taken in end-diastole, some have advocated for measurement in end-systole. There are also differences in the approach to body size adjustment, with body surface area, body mass index, and height all used in current practice.

Important work has been conducted to generate meta-analysis results of reference ranges for use in clinical practice [1]. While important and clinically useful, these contributions are limited by substantial heterogeneity between source studies [2]. Recent efforts have attempted to create reference ranges in large cohorts using standardized post-processing software and image analysis protocols. The Canadian Alliance for Healthy Heart and Minds [3] and The Healthy Hearts Consortium [4], [5] are notable examples. The latter provides reference ranges derived using different established segmentation and body size adjustment methods, so that clinical users may select their preferred option. While these contributions provide practical solutions based on current evidence, a more uniform standardization is needed to ensure global cross-comparability of CMR metrics.

Critically, the clinical utility of reference ranges depends on their ability to discriminate and predict outcomes. The absence of appropriate datasets has, until now, limited evaluation of the prognostic value of CMR reference ranges.

The Multi-Ethnic Study of Atherosclerosis (MESA) is a large prospective cohort initiated in 2000, enrolling approximately 6800 men and women aged 45–84 years, without cardiovascular disease at baseline [6]. CMR imaging was performed for over 5000 participants, acquiring cine gradient-echo images, including short-axis stacks plus two- and four-chamber long-axis views. Prospective outcome ascertainment is conducted through a rigorous, standardised process to ensure accurate capture of cardiovascular events. Thus, the cohort is an ideal platform for prognostic validation of CMR reference ranges, providing a large sample of CMR scans in healthy individuals with long-term prospective outcome tracking.

Kawel-Boehm et al. [7] present an important first step towards prognostic validation of CMR reference ranges. The authors defined conventional ventricular structure and function metrics in 4912 MESA participants with adequate CMR data available. From these, they defined sex-specific reference ranges among 1528 individuals without any cardiovascular risk factors. They found that individuals with LV structure and function parameters deviating more than two standard deviations from the healthy reference mean had significantly greater hazard of incident cardiovascular events over five and ten years of longitudinal follow-up. Interestingly, similar deviations of right ventricular metrics from the reference range did not exhibit significant associations with incident health events – which may relate to greater measurement noise and limited heterogeneity in these metrics within the sample.

Some potential limitations warrant discussion: The MESA protocol applies the “smoothed” contouring method, defining the subendocardial contour as a user- or software-defined rounded line that excludes trabeculae and papillary muscles from the LV mass, counting both as volume. The exclusion of myocardial mass has significant implications for quantitative results for LV mass and volumes [8], [9]. Because most forms of LV hypertrophy are specifically pronounced in the trabecular layer [10], [11], [12], the anatomically incorrect approach may have masked pathology. Furthermore, mass and volume values were normalized to body surface area, a commonly used approach. Echocardiography studies from the Framingham Heart Study however found a stronger prognostic value of LV mass and volume when indexed to height than when indexed to body surface [4], suggesting that height adjustment may be more suitable in a population that includes individuals with obese body shapes [5].

Further studies evaluating different approaches to reference range creation are needed to establish global consensus and standardization guidelines. Considering population diversity and technical factors associated with image acquisition and analysis, it may be necessary to establish multiple reference standards tailored to specific contexts. These efforts should be informed by understanding predictive associations of CMR metrics derived using the various clinically used methods in sufficiently large cohorts with prospective follow-up.

The increasing availability of prospective cohorts with large-sale CMR imaging – such as MESA, UK Biobank, and the German National Cohort – provide valuable opportunities to develop widely applicable, outcome-driven CMR reference standards. Collaborative, international efforts that prioritize both technical standardization and clinical utility will be key to establishing CMR reference ranges that serve diverse populations and support precision cardiovascular medicine.

The key contribution from Kawel-Boehm et al. [7] is demonstrating the prospective association of CMR reference thresholds with clinical outcomes, which provides more valuable information than the mere classification as normal or abnormal. While we will still need to assess whether the clinical application of results with additional prognostic information eventually leads to better outcomes, projects like the one reported here further improve our understanding of cardiovascular disease and help translate CMR derived phenotyping to better support clinical decision-making.

## Declaration of interests

The authors declare that they have no known competing financial interests or personal relationships that could have appeared to influence the work reported in this paper.

## Disclosures

The authors have nothing to disclose.
